# *Syngamus trachea* in free-ranging white stork (*Ciconia ciconia*) nestlings in Switzerland

**DOI:** 10.1016/j.ijppaw.2022.04.007

**Published:** 2022-04-21

**Authors:** Seraina L. Meister, Christian Wenker, Fabia Wyss, Irene Zühlke, Inês Berenguer Veiga, Walter U. Basso

**Affiliations:** a Institute of Parasitology, Department of Infectious Diseases and Pathobiology, Vetsuisse Faculty, University of Bern, Länggassstrasse 122, CH-3012, Bern, Switzerland; b Zoo Basel, Binningerstrasse 40, CH-4054, Basel, Switzerland; c Institute of Animal Pathology, Department of Infectious Diseases and Pathobiology, Vetsuisse Faculty, University of Bern, Länggassstrasse 122, CH-3012, Bern, Switzerland

**Keywords:** White stork, *Ciconia ciconia*, Parasite, *Syngamus trachea*, *Toxoplasma gondii*, PCR

## Abstract

Syngamosis is a disease caused by the strongylid nematode *Syngamus trachea,* which infects the respiratory tract of various bird species around the world. The parasite appears to be harmful for a wide variety of avian orders, occasionally leading to a fatal outcome, particularly in young birds.

The aim of this study was to examine the parasitic fauna in deceased or euthanized, free-ranging white storks nesting at the Zoo Basel in 2019 and 2020; and to assess the extent to which these parasites contributed to the wild birds' death.

In five out of 24 necropsied white storks, an infection with *S. trachea* was diagnosed based on morphological analysis of adult nematode stages and eggs, in combination with PCR amplification and sequencing of DNA extracted from female worms. The main pathological changes affected the white storks’ respiratory tract and a mixed cell tracheitis was diagnosed in the histopathological examination of three of the five infected birds. Some birds displayed additional lesions compatible with syngamosis, namely partially degenerated parasitic structures with concurrent granulomatous inflammation in the lung and multifocal acute hemorrhages in the bronchi and parabronchi. Coprological examinations (fecal flotation technique, fecal sedimentation technique, sodium acetate acetic acid formalin procedure and Ziehl-Neelsen staining) from the intestinal content as well as a PCR for *Toxoplasma gondii* on brain, lung, heart, liver, and spleen tissue yielded negative results in all examined individuals. In the absence of further major pathological findings, *S. trachea* was assumed to have significantly contributed to the death of the infected birds.

## Highlights

• 5 out of 24 dead, free-ranging white storks were infected with *S. trachea.*
• All infected white storks were nestlings.
• 3 of the 5 infected nestlings displayed a mixed cell tracheitis.
• 120 tissue samples from 24 free-ranging white storks were negative for *T. gondii.*

## Graphical abstract

**Figure:**
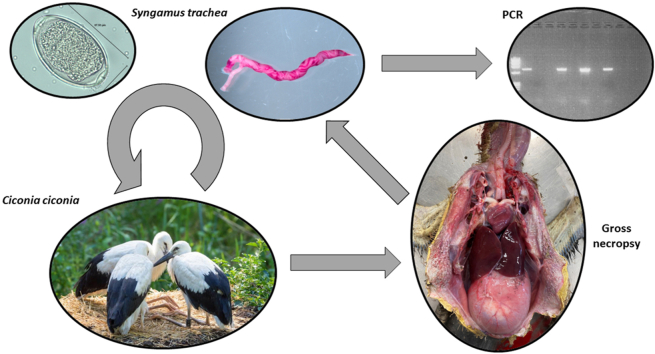

## 1 Introduction

*Syngamus trachea* (tracheal worm) is a strongylid nematode belonging to the family Syngamidae that infects the respiratory tract of a wide variety of bird species. Given its bright red color and the fact that severely affected individuals typically show a gaping-beak posture, the parasite is also known as redworm or gapeworm.

The tracheal worm's transmission occurs either directly through ingestion of the infective third-stage larvae (L3) in embryonated eggs or already hatched as free-living L3 by the bird; or indirectly, by ingestion of paratenic hosts like earthworms, snails, slugs, and insects. Infected birds harbor the adult stages of the parasite within their trachea, where the male and female worms form the typical “Y”-shaped configuration being locked in permanent copulation. Fertilized eggs are released by the females after a prepatent period of around 12–21 days (Deplazes et al., 2021). Eggs are either coughed up, swallowed by the bird, and excreted with the feces or directly expelled from the trachea. The eggs' embryonation takes two to four weeks and leads to the development of infective larvae (L3) (Deplazes et al., 2021). The consumption of embryonated eggs or already hatched larvae by paratenic hosts is followed by the release of the larval stages and encystation within the invertebrates' body. Upon ingestion by the avian host, the larvae penetrate the intestinal wall, enter either the coelomic cavity or the bloodstream and are carried to the lungs. After further development within the lung tissue, the worms migrate via the bronchi to the trachea, where the adult individuals reside for sexual reproduction.

While numerous infected birds do not show any clinical manifestations, signs of respiratory distress consisting of widely opened beak, gasping for air with outstretched neck, coughing, sneezing, head shaking, and anemia can be observed in severely affected individuals. The severity of the clinical signs depends on the parasite load, size, and age of the host. High burdens of *S. trachea* can lead to refusal of food and water intake, followed by rapid deterioration and death.

In the present study, 24 deceased or euthanized, free-ranging, white storks (*Ciconia ciconia*) found on the grounds of the Zoo Basel were examined in 2019 and 2020.

The aim of the study was to analyze the parasitic fauna of this white stork population, and to assess the extent to which the parasites contributed to the wild birds' death.

Since storks have a diverse diet (small mammals, large insects, amphibians, snakes, lizards, earthworms, fish, eggs and hatchlings of ground-nesting birds, mollusks, and crustaceans), and these preys often harbor parasites, we hypothesized that parasitic infections may play an important role in this bird species.

## 2 Materials and methods

### 2.1 White storks at Zoo Basel

Around 50 free-ranging white storks from about 25 breeding pairs hatch at the Zoo Basel every year. The adults live in seasonal monogamy and normally return to their nesting place of the previous year. The breeding time lasts from the beginning of April to the end of July and varies between 30 and 34 days. White storks lay three to five eggs, which are incubated by both parents. In the first weeks after hatching, the nestlings are usually protected by one parent while the other one is foraging in the close environment. The adults regurgitate the food in the nest, which is then ingested by the juveniles. At the age of six to eight weeks, the white stork nestlings start with first flight attempts.

### 2.2 Cases

In this study, 24 dead, free-ranging white storks (22 nestlings, 1 fledgling, 1 adult) from the Zoo Basel were examined in 2019 and 2020. Twenty nestlings were found dead under different white storks’ eyries, one nestling was euthanized during the yearly ringing operation due to a malformed beak, one fledgling was euthanized due to a severe injury in the chest that partially exposed the carina; and the mature white stork was euthanized due to a fracture of the distal tarsometatarsus. One nestling was found hanging down from the nest due to entanglement in a plastic cord and was salvaged by the fire department. The bird was kept inside overnight and force-fed with fish but was found dead the next day.

### 2.3 Necropsy, histopathological and parasitological examination

All individuals were dissected at the Zoo Basel and examined for ecto- and endoparasites. If parasites were found, they were washed using isotonic NaCl solution, stored in ethanol 70% and analyzed by stereomicroscope and scanning electron microscopy (SEM) as previously indicated (Sist et al., 2021).

Intestinal contents and samples of internal organs (brain, lung, heart, liver, spleen) were collected for subsequent coprological examination and analysis for *T. gondii* infection, respectively. Further tissue samples of various organs (i.e. trachea, lung, heart, liver, kidney, proventriculus, ventriculus (gizzard), small and large intestine, bursa of Fabricius) were sampled at necropsy, fixed in 4% buffered formalin and sent to the Institute of Animal Pathology at the University of Bern, where they were processed for routine histopathological examination by performing paraffin embedding, cutting at 2–3 μm with a semi-automatic rotary microtome, mounting on glass slides, and staining with hematoxylin and eosin (H&E).

### 2.4 Coprological examination

Intestinal contents were collected from each individual during necropsy and analyzed using the following routine diagnostic techniques: fecal flotation technique, fecal sedimentation technique, sodium acetate acetic acid formalin (SAF) procedure and Ziehl-Neelsen (ZN) staining (Deplazes et al., 2021). Microscopic examination was performed at 100x magnification for sedimentation and at 200x and 400x magnifications for flotation and SAF.

### 2.5 DNA extraction

#### 2.5.1 Syngamus trachea identification

DNA was extracted from a total of five female *S. trachea* worms (one worm per infected stork). Prior to the DNA extraction, the worms were washed using isotonic NaCl solution. A piece of approximately 3 mm length was excised from the female worms' body and dried at room temperature for at least 2 h. The DNA was then extracted from the tissue using the Spin-Column Protocol for animal tissues from the DNeasy® Blood & Tissue Kit (QIAGEN GmbH, Hilden, Germany) according to the manufacturer's protocol.

#### 2.5.2 Internal organs for Toxoplasma gondii detection

DNA from the white storks' internal organs (brain, lung, heart, liver, spleen) was isolated using the Spin-Column Protocol for animal tissues from the DNeasy® Blood & Tissue Kit (QIAGEN GmbH, Hilden, Germany) with the following modification (Berger-Schoch et al., 2011): 500 μg tissue were mixed with 100 μl proteinase K and 900 μl ATL buffer, followed by an over-night incubation at 56 °C; 200 μl of the digested material were then further processed according to the manufacturer's instructions.

### 2.6 PCR

#### 2.6.1 Syngamus trachea molecular analysis

A PCR targeting the mitochondrial cytochrome *c* oxidase subunit I gene (*cox-1*) was performed (Casiraghi et al., 2001). One primer set (Microsynth AG, Balgach, Switzerland) was used at a 100 μM working solution. Per tube, 0.25 μl of each primer (COIintF and COIintR) were mixed with 12.5 μl of a commercial master mix (QIAGEN Multiplex PCR Kit, Hombrechtikon, Switzerland), 9.5 μl ddH_2_0 and 2.5 μl of DNA template to a total volume of 25 μl. The PCR amplification was performed under the following conditions: 95 °C for 15’ (for activation of HotStarTaq DNA Polymerase), followed by 40 cycles of denaturation at 94 °C for 45″, annealing at 52 °C for 45″ and extension at 72 °C for 90″ with a final extension at 72 °C for 10’. A known positive DNA control and a negative control (ddH_2_O) were used for each run. Amplification products were resolved by gel electrophoresis in 1.5% agarose gels containing ethidium bromide for 35 min at 120 V. DNA was recovered from the PCR products using the Zymo Research Purification Kit (DNA Clean & ConcentratorTM-5, Zymo Research Europe GmbH, Lucerne, Switzerland). Subsequently, a bidirectional sequencing of the obtained amplicons was performed with the same primers used in the PCR reaction (Microsynth AG, Balgach, Switzerland), followed by comparison with sequences on GenBank® using the Nucleotide BLAST algorithm (http://blast.ncbi.nlm.nih.gov/Blast.cgi). The obtained DNA sequences were deposited in GenBank® after trimming the primer-binding regions (accession no. OM831258 - OM831262).

#### 2.6.2 Toxoplasma gondii DNA detection

A real-time PCR targeting the B1 gene (126 bp) of *T. gondii* was performed (Costa et al., 2000) on a LightCycler® Instrument (Roche Diagnostics, Basel, Switzerland). One primer set (23-mer, sense: 5′-GGAGGACTGGCAACCTGGTGTCG-3′ and 25-mer, antisense: 5′-TTGTTTCACCCGGACCGTTTAGCAG-3′) was used at a 20 μM working solution as well as a pair of LightCycler® hybridization probes (5′-ACGGGCGAGTAGCACCTGAGGAGAT-3′ and 5′-CGGAAATAGAAAGCCATGAGGCACTCC-3′) at a 2.5 μM working solution. The amplification mixture consisted of 0.25 μl of each primer, 1.0 μl of each probe, 1.0 μl of a commercial master mix (LightCycler® DNA Master Hybridization Probes Kit, Roche Diagnostics), 0.8 μl MgCl_2_ (25 mM), 4.575 μl H_2_O and 1 μl of template DNA in a final volume of 10 μl. Carry-over contamination was prevented by using 0.125 μl of heat-labile uracyl-DNA-glycosylase (UDG) per tube. The samples were amplified using the following thermal profile: 40 °C for 10’ (to allow the UDG to act), 95 °C for 15’ (for denaturation of the template DNA, inactivation of the UDG enzyme and activation of the FastStart Taq DNA Polymerase), followed by 50 cycles of denaturation at 95 °C for 10″, annealing at 56 °C for 20″ and extension at 72 °C for 20″. A known positive DNA control and a negative control (ddH_2_O) were used for each run.

## 3 Results

The parasitological examination revealed an infection with *S. trachea* in five of the 24 analyzed individuals in 2019 and 2020. The diagnosis was based on the morphological analysis of adult stages and eggs, in combination with PCR amplification and sequencing of DNA extracted from female worms.

### 3.1 Morphology of adults and eggs

**Fig. 1:**
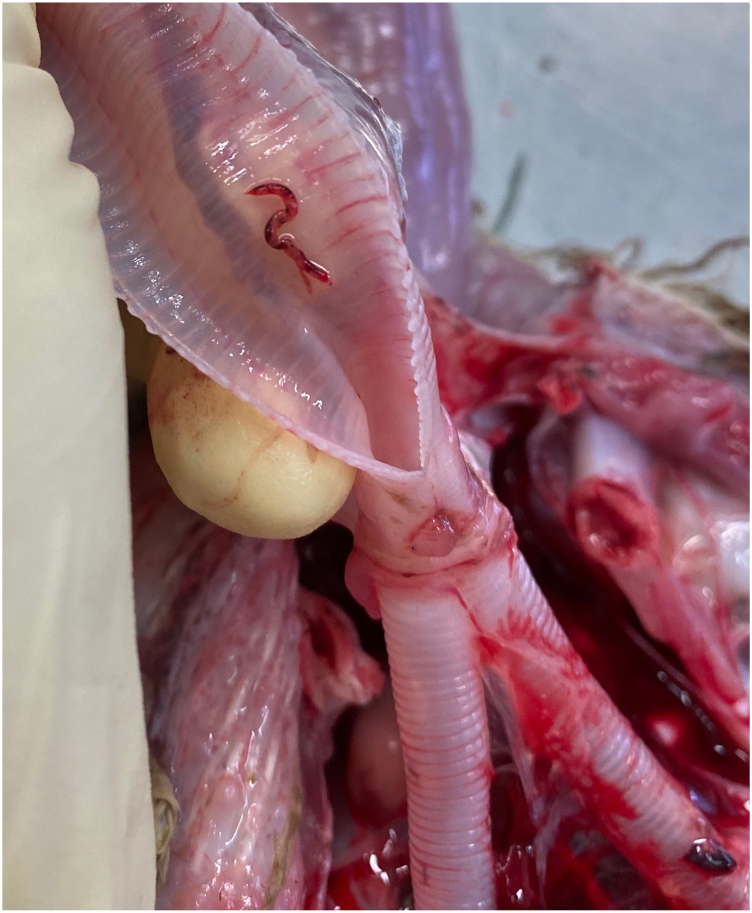
Necropsy of a white stork nestling (WS3) showing the opened trachea with *Syngamus trachea* forming the typical Y-shape.

**Fig. 2:**
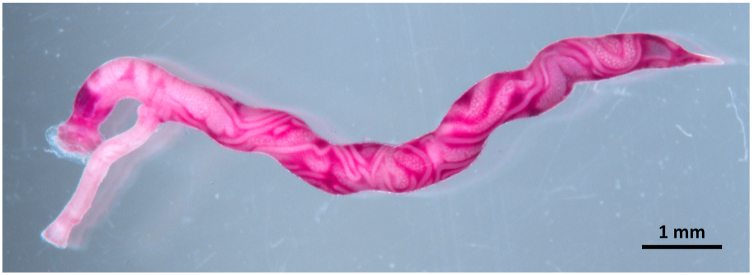
Stereo microscope image of a *Syngamus trachea* pair locked in permanent copulation.

**Fig. 3:**
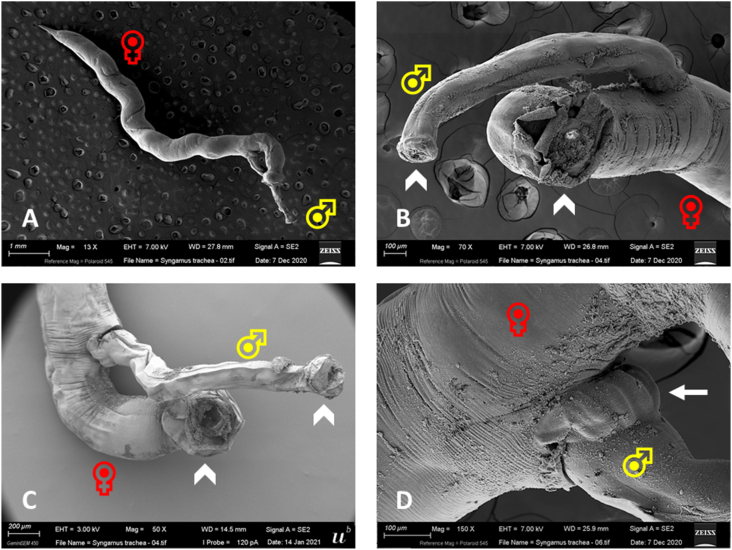
SEM image of a female and male *Syngamus trachea* (A), with a large cup-shaped buccal capsule (indented arrows) in both adult nematodes (B, C) and a prominent but short copulatory bursa (arrow) in the male individual (D).

Numerous bright red adult nematodes (Fig. 1), forming the typical “Y” shape (Fig. 2, Fig. 3A), with a large cup-shaped buccal capsule (Fig. 3B and C) were found in the trachea of the five infected white stork nestlings at necropsy. The females measured between 5 and 10 mm and the attached males between 3 and 6 mm, showing a prominent but short copulatory bursa (Fig. 3D).

**Fig. 4:**
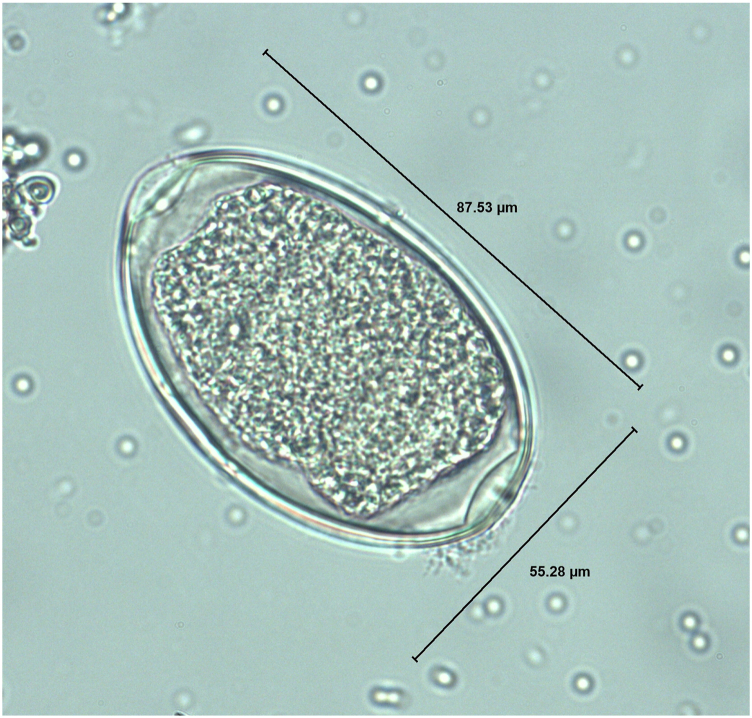
Egg of *Syngamus trachea*: ellipsoidal, with distinct bipolar opercula, 87.53 μm long and 55.28 μm wide.

Numerous eggs were directly extracted from the females’ uterus. They were between 81 and 93 μm long and 41–56 μm wide, ellipsoidal, morulated, and with distinct bipolar opercula (Fig. 4). No eggs were detected using coprological examinations (fecal flotation technique, fecal sedimentation technique, SAF and ZN staining) of the intestinal content of all 24 animals.

### 3.2 PCR analysis

**Fig. 5:**
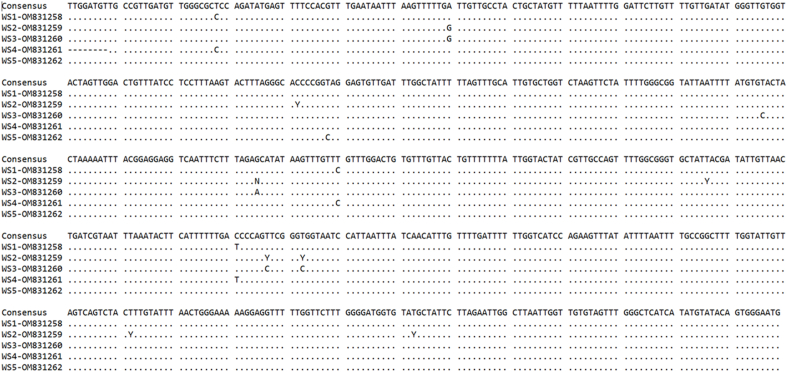
Haplotypes from female *S. trachea* nematodes collected from five different white stork nestlings (WS1-5).

All five PCR products (WS1-5) obtained from the female worms showed the expected band size of 688 bp, and the obtained sequences were compared with one another (Fig. 5). Case one (WS1: OM831258) and four (WS4: OM831261) were identical to each other except for the missing first eight bases at the 5’ end in the latter. Case five (WS5: OM831262) corresponded most closely to case one (WS1: OM831258) and four (WS4: OM831261) with four bases being different between them. Case two (WS2: OM831259) showed four clear base differences and seven differences due to ambiguous (Y or N) nucleotide identification in comparison to case one (WS1: OM831258) and four (WS4: OM831261). Case 3 (WS3: OM831260) corresponded most closely to case five (WS5: OM831262) with a difference of six bases.

In all five cases (WS1-5), the sequences shared the highest BLAST identity with a GenBank® sequence of *S. trachea* collected from an adult Australian Magpie (*Gymnorhina tibicen*) in Victoria, Australia (GQ888718) (Jex et al., 2010). In case one (WS1: OM831258) and three (WS3: OM831260), the BLAST identity with the above-mentioned sequence was 99.23% (644/649 bp), in case two (WS2: OM831259) 98.92% (642/649 bp), in case four (WS4: OM831261) 99.22% (636/641 bp) and in case five (WS5: OM831262) 99.54% (646/649 bp).

The PCR for *T. gondii* of the white storks’ internal organs yielded negative results for all 120 tissue samples (brain, lung, heart, liver, spleen) of the 24 examined animals.

### 3.3 Histopathology

**Fig. 6:**
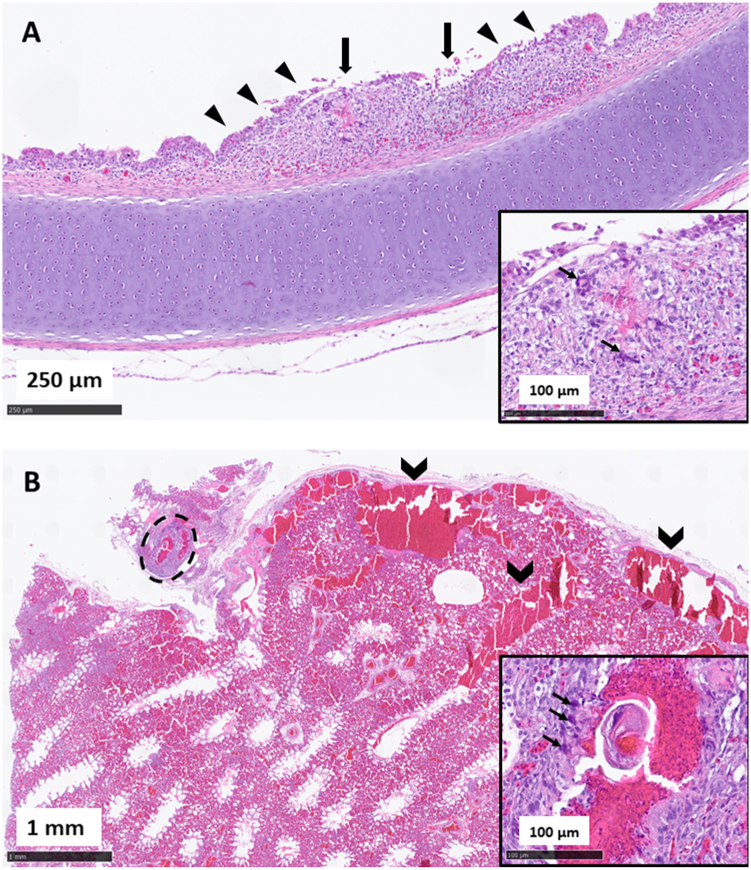
Histopathological lesions observed in the respiratory tract of one of the affected animals from this study. Focal-extensive erosion and ulceration of the tracheal mucosa (large arrows) with thickening of the lamina propria (arrowheads) due to a mixed cell tracheitis and focal granuloma formation in animal WS3 (A). The inset shows a larger magnification of this granuloma, which consists of a necrotic core surrounded by few multinucleated giant cells (narrow arrows), macrophages, and heterophils. H&E staining. Partially degenerated parasitic structure within the lung parenchyma (dashed line) with multifocal acute hemorrhages (indented arrows) in the bronchi and parabronchi of animal WS3 (B). The inset shows a larger magnification of the degenerated parasite with surrounding granulomatous inflammation, composed of abundant multinuclear giant cells (narrow arrows), macrophages, heterophils and acute hemorrhage. H&E staining.

The main histopathological findings affected the white storks’ respiratory tract. Three of the five birds infected with *S. trachea* displayed a mixed cell tracheitis, characterized by a diffuse, moderate lymphocytic, plasmacellular and heterophilic infiltration of the tracheal mucosa. In one case (WS3), the trachea was additionally focally nodular enlarged due to the presence of a small granuloma (Fig. 6A) consisting of a necrotic, eosinophilic center surrounded by few multinucleated giant cells, macrophages and heterophils (Fig. 6A, inset). Also, the bronchi and parabronchi of this bird were multifocally filled with extravasated erythrocytes (Fig. 6B). Additionally, a partially degenerated parasitic structure of approximately 50 μm in diameter surrounded by a granulomatous reaction (Fig. 6B), composed of multinucleated giant cells, macrophages and heterophils (Fig. 6B, inset), was detected in the lung parenchyma (parabronchus) of this animal. In another case (WS5), there was a focal, granulomatous, interstitial pneumonia, characterized by the presence of multinucleated giant cells, heterophils, lymphocytes and cell debris surrounding few central, PAS-positive, suspicious fungal structures. This animal also displayed a moderate, acute, heterophilic enteritis and cloacitis.

## 4 Discussion

This case series describes the diagnosis of *S. trachea* and associated histopathological changes in deceased, free-ranging white stork nestlings found at Zoo Basel in 2019 and 2020.

The exact age of the deceased white stork nestlings was unknown, but the estimated age was between two and six weeks. All infected white storks were nestlings that had not acquired flight capability at the time of death. Thus, we assume that the birds got infected by contaminated food and/or paratenic hosts, which had been regurgitated by the parents to feed their offspring. In another study from 2020 (Michalczyk et al., 2020), adult stages of *S. trachea* were found in two out of 38 examined white storks. Both affected individuals were three to four weeks old and tracheal worms could not be detected in older storks in that study. We presume that the immune system of the analyzed white stork nestlings has not fully developed yet, thus increasing the risk for a parasitic infection as well as a lethal outcome post-infection. Immune immaturity in the first three weeks after hatching has been proposed as major predisposing factor for other diseases in white storks (Olias et al., 2010, 2011; Jovani and Tella, 2004). The susceptibility of young birds may also explain why most syngamosis cases occur in the spring to summer months in association with the breeding cycles of white storks as previously described (Nevarez et al., 2002; Cole, 2001). Accordingly, the prevalence of infection with *S. trachea* decreases considerably in free-living populations as birds mature, suggesting that most natural hosts acquire resistance against the parasite (Fernando and Barta, 2008). Not only the risk for infection is higher in juveniles, but also the risk of developing more severe clinical signs. Smaller-sized and younger birds have narrower airways and are therefore more prone to luminal obstruction due to the adult worms’ growth in the trachea and consequent mucus production (Nevarez et al., 2002). Since the examined individuals in this study were free-ranging and white storks build their eyries in considerable height, the nestlings could not be regularly monitored; therefore, it is unclear if clinical signs occurred prior to death.

Within the trachea, the parasites cause direct mechanical damage to the trachea's mucosa (erosions and ulcerations) and produce hemorrhages originating from their blood meals. Host-mediated inflammatory responses to these mechanical lesions (tracheitis, granuloma formation), and possibly parasite antigen and/or secretory products exacerbate this mechanical damage (Fernando and Barta, 2008). The histopathological findings in this study resemble those found in other avian species and reinforced the suspicion of an infection with *S. trachea* based on gross pathology (Martin et al., 2020; Fernando and Barta, 2008). In the presumptively oldest infected nestling (WS5), the histopathological examination revealed a focal granulomatous interstitial pneumonia with intralesional, suspicious fungal structures as well as an acute heterophilic enteritis and cloacitis. The parasitic infection could have compromised this bird's health status, thereby predisposing it to further diseases.

An additional factor to consider concerning the nestlings’ cause of death is parental infanticide which is quite commonly observed in white storks. However, in this case we would expect traumatic hemorrhages at necropsy and such lesions were not observed.

The absence of *S. trachea* egg stages following routine fecal flotation from the intestinal content obtained upon necropsy may be explained by the timepoint of the analysis. According to the literature, the first eggs can be found 12–21 days post infection (Deplazes et al., 2021), therefore we assume that we analyzed the intestinal contents right before the prepatent period ended since morulated eggs were present within the females’ uterus but not yet in the coprological examination.

Effective anthelmintics against *S. trachea* exist, but there is no feasible method for controlling tracheal worms in free-ranging birds. Deworming of the white stork nestlings would theoretically be possible during the ringing operations; however, in our experience the critical phase for the nestlings usually occurs earlier, mostly within the first four weeks after hatching. Consequently, routine deworming at the time point of the official ringing is not regarded as an essential intervention. Since the nestlings are only handled in course of the ringing operations and treatment of free-ranging animals is not routinely performed at the zoo, successful prophylaxis and extensive medical treatment of the white stork nestlings will not be performed at the Zoo Basel in the future. Regarding the relatively low prevalence (five out of 24 white storks) the need for such an intervention may be questionable. The prevalence in nestlings that do not fall from the nest is not exactly known. Since only deceased and euthanized animals were analyzed for this study and clinically unremarkable individuals were not captured to specifically check for *S. trachea*, a reliable statement about the prevalence of *S. trachea* in alive, uninjured individuals cannot be made. However, no eggs of *S. trachea* were found in fecal samples of 34 nestlings (age: between 6 and 8 weeks) collected during the annual official ringing operations in 2019 and 2020 (from in total 237 nestlings from the Zoo Basel and the near environment).

Free-ranging birds shedding egg stages of *S. trachea* could theoretically infect other avian species including captive birds belonging to the zoo's collection. However, no syngamosis outbreaks have been observed so far.

The PCR for *T. gondii* was negative in all tissue samples from the 24 examined white storks. Nevertheless, the investigation of infection with *T. gondii* in free-ranging birds is a useful way to assess environmental contamination with oocysts, since different avian species feed directly on the ground and are continuously exposed to oocyst ingestion (Waap et al., 2008). In addition, another way of infection for omnivorous birds such as white storks is the ingestion of infected intermediate or paratenic hosts, and this also applies to the nestlings, which could get infected through ingestion of these hosts when regurgitated in the nest by their parents.

## 5 Conclusion

This study describes the infection with *S. trachea* in five of 24 analyzed free-ranging white storks in 2019 and 2020 based on necropsy findings, histopathological examination, morphological analysis of adult nematodes and eggs, PCR analysis and subsequent sequencing. Coprological examinations of intestinal contents as well as a PCR for *T. gondii* of the white storks’ internal organs yielded negative results. In absence of other major pathological findings, *S. trachea* was assumed to have significantly contributed to the death of the infected birds.

## Funding source declaration

This study was financially supported by the “Tierspital in Basel” Foundation, Zoo Basel and the Institute of Parasitology of Bern, Switzerland.

## Declaration of competing interest

The authors declare that they have no known competing financial interests or personal relationships that could have appeared to influence the work reported in this paper.
